# Factor Analysis of the CES-D 12 among a Community Sample of Black Men

**DOI:** 10.1177/1557988319834105

**Published:** 2019-03-20

**Authors:** Leslie B. Adams, Nisha Gottfredson, Alexandra F. Lightfoot, Giselle Corbie-Smith, Carol Golin, Wizdom Powell

**Affiliations:** 1 Department of Health Behavior, Gillings School of Global Public Health, University of North Carolina at Chapel Hill, Chapel Hill, NC, USA; 2 Center for Population and Development Studies, Harvard University, Cambridge, MA, USA; 3 Department of Social Medicine, UNC School of Medicine, University of North Carolina at Chapel Hill, Chapel Hill, NC, USA; 4 Center for Health Equity Research, UNC School of Medicine, University of North Carolina at Chapel Hill, Chapel Hill, NC, USA; 5 Department of General Medicine and Epidemiology, Department of Medicine, UNC School of Medicine, University of North Carolina at Chapel Hill, Chapel Hill, NC, USA; 6 Health Disparities Institute, University of Connecticut, Hartford, CT, USA

**Keywords:** depression, psychometrics, CES-D, factor analysis, Black men

## Abstract

The Center for Epidemiologic Studies Depression (CES-D) scale is one of the most widely used measures for assessing depression in population-based research. Little is known about the varying range of symptomatology expressed by Black men, who report higher chronicity and disability of their depressive symptoms compared to men of other racial and ethnic backgrounds. This study assessed the dimensional structure of the CES-D 12-item scale using exploratory and confirmatory factor analysis in a community-based sample of Black men (*n* = 683). Two latent factors emerged from the scale that best fit the data: interpersonal negative affect (INA) and diminished positive affect (DPA). The item “I felt like everything I did was an effort” was removed from the final measure, resulting in an 11-item scale. The total score for the revised CES-D-11 displayed acceptable internal consistency on both latent factors (Cronbach’s α = 0.83 [INA] and 0.73 [DPA]) and model fit (χ^2^ = 165.58, TLI = 0.967, CFI = 0.974, RMSEA = 0.065). Results differ from CES-D factor analyses in other demographic groups, including studies with other male subpopulations, such that depressed mood and interpersonal problems factors are merged as a unidimensional construct. Findings suggest that the “effort” item from the CES-D 12 should be interpreted with caution among Black men. Future studies should continue to disentangle the divergent pathways in which Black men express depressed mood.

The Center for Epidemiologic Studies Depression (CES-D) scale is one of the most widely used measures for assessing depression in population-based research (Radloff, 1977). Since the original 20-item scale was created, the CES-D scale has been abbreviated to 12 items to more efficiently capture depressive symptom structures and reduce respondent burden across diverse populations (Assari & Moazen-Zadeh, 2016; Kim, Decoster, Huang, & Chiriboga, 2011; Kohout, Berkman, Evans, & Cornoni-Huntley, 1993; Zauszniewski, Bekhet, & Suresky, 2009). The abbreviated scale is equivalent to the original 20-item measure in two ways; namely, the scale captures comparable dimensions of depression and demonstrates strong predictive validity to clinical diagnostic tools (Assari & Moazen-Zadeh, 2016; Torres, 2012). Methodologically, research in this field typically describes depression as a unidimensional construct, using summed scores or aggregated symptom counts to characterize the condition (Fried, Nesse, Zivin, Guille, & Sen, 2014). By utilizing approaches that capture the underlying clustering of depressive symptoms, such as factor analysis, researchers can better assess pathways to identify and treat this disorder.

Extant literature examining the CES-D factor structure among Black Americans have yielded mixed results, which suggests that the measure may not have a universally consistent dimensionality (Hertzog, Van Alstine, Usala, Hultsch, & Dixon, 1990; Kim, Chiriboga, & Jang, 2009; Kim et al., 2011; Makambi, Williams, Taylor, Rosenberg, & Adams-Campbell, 2009; Posner, Stewart, Marín, & Pérez-Stable, 2001; Roberts, 1980). Few studies examine the CES-D factor structure among Black men who, compared to men of other racial and ethnic backgrounds, experience prolonged severity and chronicity associated with depressive symptoms and alarmingly increased rates of suicidal behavior among youth (Bridge et al., 2015; Wadsworth, Kubrin, & Herting, 2014; Ward & Mengesha, 2013; Williams, 2003). In the past decade, the psychometric properties of the 12-item measure were assessed in a single study in 2012 using a nationally representative sample of Black men (Torres, 2012). Yet, factor structure using the 12-item scale remains unexplored in study samples comprised of Black men. Given that Black men seek health services for mental health issues at lower rates than the general population, capturing depressive symptomatology in a community-based setting is of added importance for future research aimed at fostering mental well-being within this population (Ward & Mengesha, 2013).

With some notable exceptions, literature assessing the dimensionality of Black men’s depressive symptomatology, as characterized by the CES-D, is sparse. An exploratory factor analysis (EFA) study conducted by Callahan and Wolinsky (1994) reported considerable variation in the original 20-item CES-D factor structure among elderly Black male patients compared to other race–gender groups in the primary care setting. This study determined seven underlying CES-D factors and introduced emerging factors that are not historically reflected in factor analysis literature, including anxiety, introspection, and crying. Another exploratory study by Love and Love (2006) identified three emerging factors in a sample of older Black men residing in Harlem, with the depressive and somatic factors merging into a single factor of depression among Black men. Both authors concluded by recommending future research to examine depression factor scores among Black men in broader community-based settings to determine the extent to which sociocultural differences influence item endorsement and the underlying clustering of depressive symptoms in the general population.

This manuscript addresses the previously noted gap by identifying the appropriate factor structure among a larger, community-based sample of socioeconomically diverse Black men. First, an EFA was conducted to examine dimensional structure and allow for openly guided evaluation of item loading and factor structure. Second, using the EFA-implied solution, the retained structure was verified using a confirmatory approach. Guided by previous studies, the central hypothesis is that depressive symptoms, measured through the 12-item CES-D, will cluster along three correlated latent factors: positive affect, negative affect, and interpersonal problems.

## Methods

### Data Source

Data were collected from the African American Men’s Health and Social Life (AAMHSL) study, a cross-sectional, community-based survey study conducted between 2007 and 2011. The goal of the AAMHSL study was to assess a range of attitudes, behaviors, and health status of Black men residing in the United States. The questionnaire assessed men’s early life health care experiences, religious/spiritual values, experiences with daily stress (general and race related), gender norms, and current health care utilization practices. Convenience sampling methods were used in the AAMHSL to recruit a sample of Black men from various academic and community settings. Most participants (*n* = 551, 80.7%) were recruited from barbershops in Michigan and Georgia. The remainder of the study participants (*n* = 132, 19.3%) were recruited from academic institutions and events, which included a conference for Black men sponsored by a professional labor association. The academic institutions were a community college located in Southeastern Michigan and a historically Black colleges and universities (HBCU) institution in central North Carolina.

Black men age 18 years and older were recruited through a variety of means, including flyer advertisements, direct contact, word of mouth, specially advertised data collection events, and e-mail solicitation. Men who expressed interest in the survey were directed to study personnel. Before the study proceeded, informed consent was obtained through verbal and written documentation, and the anonymous self-administered survey was administered. Additional information regarding the data collection procedure is detailed in previous AAMHSL studies (Hammond, Matthews, & Corbie-Smith, 2010; Matthews, Hammond, Nuru-Jeter, Cole-Lewis, & Melvin, 2012).

### Procedures

Barbershops with a high customer volume were prioritized as recruitment sites for two primary reasons. First, long wait times could be used as time required to complete the questionnaire by study participants. Second, extant literature suggests that barbershops are typically patronized by a socioeconomically diverse group of Black men (Hart & Bowen, 2004). Initial contact with barbershops was made in person or by telephone by study personnel. Barbers or receptionists, not study personnel, invited men to complete the questionnaire based on previous empirical support positioning these individuals as trusted community stakeholders and integral to community-engaged research in Black communities (Cowart, Brown, & Biro, 2004; Hammond, et al., 2010; Releford, Frencher, & Yancey, 2010). Men provided verbal and written consent to study staff at the beginning of the study and those who completed the questionnaire received a voucher for a free haircut, valued at $25. As an incentive for its participation in the study, the barbershop retained any unused value of the voucher. In addition to barbershops, participants were recruited from two academic settings, one historically Black and the other predominantly White. Recruitment methods were similar at these sites, with the exception that study personnel rather than barbershop staff recruited Black men directly. Study participants were also encouraged to spread the word about the research team’s presence and invite their peers to complete a questionnaire. The research team solicited study participation in high-traffic areas such as the student union or eating areas at academic institutions. Participants recruited at these sites who participated also received a $25 gift card. All study procedures were reviewed and approved by the Institutional Review Board at the University of North Carolina at Chapel Hill.

### Measures

#### Depressive symptomatology (CES-D, 12-item)

Depressive symptomatology was measured using the 12-item version of the CES-D scale (Radloff, 1977). Categorical response variables for each item ranged from 0 (*Rarely or none of the time*) to 3 (*Most or all of the time*). Items that reflected a more positive mood (e.g., “I was happy”) were reverse coded to reflect higher depressive symptomatology.

#### Demographics

Demographic characteristics measured in the study that were used in this analysis included age, measured continuously, education status (e.g. some high school to graduate and professional school), and employment status (e.g. full-time, part-time, student, or unemployed).

### Analysis

Data management and descriptive statistics were conducted in SPSS 24.0. EFA and CFA were conducted in Mplus version 8.0 (Muthén & Muthén, 1998–2018). Four positive affect CES-D items were reversed coded so that all higher scores reflected more depressive symptomatology. Univariate analyses were conducted for all study variables to assess violations of the normality assumption for item response distributions. Bivariate analyses of the CES-D items and total CES-D score were also conducted to determine inter-item and alpha reliability.

Due to inconsistent factor structure findings and limited investigations among Black men in the existing literature, an EFA was first conducted to establish a recommended factor structure that would be tested in the confirmatory phase of the analysis. To ensure generalizability in our sample, the full AAMHSL dataset (*n* = 683) was randomly split into two halves to conduct the exploratory and confirmatory analyses, respectively. The benefit of this approach is twofold. First, the split samples can be used to validate and cross-check findings using a single demographic sample. Second, this approach provides further insight into scale stability (DeVellis, 2016). The EFA was conducted on the first half of the randomly split sample using varimax rotation. We tested a one-, two-, and three-factor model to determine the appropriate number of factors to retain the criteria described by DeVellis (2016). Higher factor loadings for each CES-D item were used to signal the primary factor where the item would be loaded.

Second, we conducted the CFA using the remaining randomly split sample. We confirmed the fit of the retained EFA model as well as the previously identified three-factor structure that is recommended in the literature (Assari & Moazen-Zadeh, 2016). We assessed model fit using a weighted least squares estimator (WLSMV) to account for the categorical nature of the CES-D scale. Model fit was determined as acceptable by goodness-of-fit indices including the chi-square (χ^2^), comparative fit index (CFI > 0.95), Tucker–Lewis index (TLI > 0.95), root-mean square error of approximation (RMSEA ≤ 0.06), and modification indices based on cutoff values for the fit indices primarily specified by Hu and Bentler (1999).

## Results

### Descriptive Statistics

Table 1 illustrates the descriptive characteristics of all AAMHSL study participants (*n* = 683). Overall, the mean age was 32 years and ranged between 18 and 79 years. Most participants received a high school education or greater and were employed full-time. The average CES-D score was 11.18, with a range between 0 and 25 on the 12-item CES-D scale. Descriptive analysis, including means, standard deviations, and item-to-total correlations for each CES-D item, is shown in Table 2. Three items had item-to-total correlations at or below 0.40: “I felt that everything I did was an effort” (*r* = .04), “I felt that I was just as good as other people” (*r* = .27), and “I felt hopeful about the future” (*r* = .22). The internal consistency for the overall 12-item CES-D was high (α = .78). Responses were skewed toward low-to-moderate depressive symptomatology such that men were more likely to report experience symptoms none of the time, some of the time, or occasionally.

**Table 1.** Descriptive Statistics of AAMHSL Study Participants (*n* = 683).

Variable	*n* (%) or mean [*SD*]
Age, years	32.18 [11.18]
Min, max	18, 79
Education	
Less than high school	25 (3.7)
High school	192 (28.1)
Some college	225 (32.9)
College degree	127 (18.6)
Graduate or professional degree	55 (8)
Employment status	
Full time	408 (60)
Part time	78 (11.5)
Unemployed	122 (17.9)
Student	72 (10.6)
CES-D score	11.18 [5.87]
Min, max	0, 25

*Note.* AAMHSL = African American Men’s Health and Social Life study; CES-D = Center for Epidemiologic Studies Depression scale.

**Table 2.** Sample Mean, Item-to-Total Correlations, and Response Distributions for AAMHSL Participants (*n* = 683).

CES-D item	Mean (*SD*)	Item–total correlation	Response categories (%)			
			Rarely	Some of the time	Occasionally	Most of the time
I felt that I was just as good as other people^a^	0.86 (0.97)	0.27	7.1	19.3	26.2	47.4
I had trouble keeping my mind on what I was doing	1.12 (0.93)	0.45	31.2	32.2	29.7	6.8
I felt depressed	0.80 (0.92)	0.68	49.2	29.0	17.0	5.8
I felt that everything I did was an effort	1.66 (1.02)	0.04	16.4	25.7	33.6	24.3
My sleep was restless	1.14 (0.97)	0.42	31.5	31.8	27.6	9.2
I was happy^a^	0.91 (0.90)	0.46	6.4	16.9	37.7	38.9
People were unfriendly	1.09 (0.93)	0.49	30.6	38.4	22.7	8.3
I enjoyed life^a^	0.71 (0.90)	0.39	5.4	14.2	26.6	53.8
I had crying spells	0.54 (0.89)	0.61	69.0	12.9	13.5	4.6
I felt that people disliked me	0.86 (0.96)	0.55	46.8	27.9	18.3	7.1
I could not get going	0.89 (0.92)	0.59	42.1	33.2	18.4	6.4
I felt hopeful about the future^a^	0.85 (1.00)	0.22	10.1	13.2	27.5	49.0

*Note*. AAMHSL = African American Men’s Health and Social Life study; CES-D = Center for Epidemiologic Studies Depression scale.

a Reverse coded to reflect higher depressive symptomatology.

### EFA Results

One-, two-, and three-factor EFA models were tested on the randomly split-half sample (*n* = 341) to determine the optimal number of factors to retain for confirmatory analysis. The one-factor EFA model yielded the poorest overall model fit (CFI = 0.809, TLI = 0.767, RMSEA = 0.162), while the two- (CFI = 0.985, TLI = 0.977, RMSEA = 0.051) and three-factor model (CFI = 0.993, TLI = 0.986, RMSEA = 0.039) demonstrated acceptable fit. The two-factor model was selected for optimal fit due to higher factor loadings and fewer cross-loaded items compared to the three-factor model. Moreover, the eigenvalue for the three-factor model (0.806) was lower than optimal EFA fit criteria retention detailed in extant research (DeVellis, 2016).

Continuing with the two-factor model, item characteristics of each retained factor were examined. Factor 1, which included eight CES-D items, reflected items related to negative affect, depressed mood, and interpersonal challenges. This factor is represented as *interpersonal negative affect (INA)*. Second, Factor 2 was comprised of four CES-D items related to positive affect. This factor is represented as *diminished positive affect (DPA)*. Table 3 displays the factor loadings from the exploratory analysis. Notably, “I felt that everything I did was an effort” demonstrated the poorest overall factor loading (INA: 0.299; DPA: −0.468) and significant loading values on both the INA and DPA factors.

**Table 3.** Standardized Factor Loadings of the Exploratory Analytic Sample.

CES-D items	Factor 1: interpersonal negative affect	Factor 2: diminished positive affect
1. I felt that I was just as good as other people	–	0.719
2. I had trouble keeping my mind on what I was doing	0.667	–
3. I felt depressed	0.776	–
4. I felt that everything I did was an effort	0.299	−0.468
5. My sleep was restless	0.617	–
6. I was happy	–	0.762
7. People were unfriendly	0.674	–
8. I enjoyed life	–	0.751
9. I had crying spells	0.757	–
10. I felt that people disliked me	0.692	–
11. I could not get going	0.740	–
12. I felt hopeful about the future	–	0.632

*Note.* CES-D = Center for Epidemiologic Studies Depression scale.

### CFA Results and Factor Loading

Table 4 summarizes confirmatory model fit indices of the CES-D items in the second split half of the AAMHSL dataset (*n* = 342). For the confirmatory approach, three CFA models were tested. Model 1 tested the hypothesized three-factor confirmatory structure, which suggested the 12 items would load onto three correlated factors, positive affect (4 items), negative affect (6 items), and interpersonal problems (2 items). This model yielded poor fit (WLSMV χ^2^ = 207.687, TLI = 0.916, CFI = 0.935, RMSEA = 0.092). Model 2 was guided by EFA results, which suggested that the “effort” item load simultaneously onto the INA and DPA factors. This model demonstrated acceptable fit as demonstrated by the fit indices (WLSMV χ^2^ = 123.368, TLI = 0.962, CFI = 0.970, RMSEA = 0.061). Finally, Model 3 illustrates the fit of a two-factor solution with the “effort” item removed from the overall scale. Analysis of this model was prompted by poor item-to-total correlations and weak factor loadings for the “effort” item from the EFA analysis. This model demonstrated slightly improved model fit as demonstrated by the TLI and CFI values (WLSMV χ^2^ = 165.583, TLI = 0.967, CFI = 0.974, RMSEA = 0.065). The 11-item scale also had improved subscale internal consistency with a Cronbach’s α of .73 (DPA) and 0.83 (INA), respectively. Thus, both Models 2 and 3 demonstrated acceptable fit for the overall model. To determine the best fit model, the psychometric properties of each item were considered, along with extant literature validating the CES-D in Black communities, and theoretical understandings of effort as a depressive symptom construct in the Black community. Using this process, Model 3, reflecting an 11-item CES-D scale with the “effort” item removed, provided the best fit overall in the study sample.

**Table 4.** Model Fit Indices of the Confirmatory Analytic Sample.

	Model	WLSMV χ^2^	*df*	*p* value	TLI	CFI	RMSEA (90% CI)
1	Three factors (hypothesized)	207.69	51	<.001	0.916	0.935	0.092 [0.079, 0.105]
2	Two factors (“effort” item loaded on both factors)	123.37	52	<.001	0.962	0.970	0.061 [0.470, 0.075]
3	Two factors (“effort” item removed)^a^	165.58	43	<.001	0.967	0.974	0.065 [0.055, 0.076]

*Note*. CFI = comparative fit index; RMSEA= root-mean square error of approximation; TLI = Tucker–Lewis index; WLSMV= weighted least squares mean estimator.

a Final measurement model.

**Figure 1.**
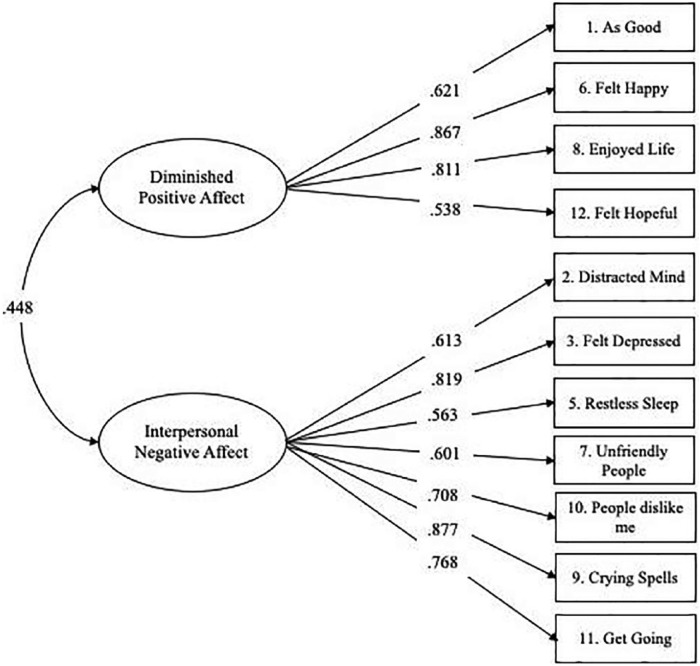
Final measurement model for depression (Center for Epidemiologic Studies Depression [CES-D] scale).

Figure 1 illustrates the final measurement model and confirmatory factor loadings of the 11-item CES-D scale. Standardized factor loadings were all statistically significant at *p* < .001 and ranged between 0.538 (“I felt hopeful about the future” on the DPA factor) and 0.877 (“I had crying spells” on the INA factor). The INA factor was comprised of seven items with factor loadings ranging from 0.563 to 0.877 and the positive factor was comprised of four items with factor loadings ranging from 0.538 to 0.867. The results of the intercorrelated model CFA also showed a significant positive correlation between the INA and DPA factors (*r* = .448, *p* < .001).

## Discussion

The current study builds on previous literature examining the factor structure of the CES-D scale by testing both an EFA and a CFA in a community-based sample of Black men. Contrary to the hypothesis, a two-factor model comprising the latent variables of INA and DPA had the best fit compared to the three-factor model found in other extant studies. Based on the results from the current study, we recommend interpreting the item “I felt like everything I did was an effort” with caution in study samples including Black men. The rationale for this recommendation is guided by both validated findings of the CFA models and theoretical considerations of effort in this community. The “effort” item exhibited poor item-to-total correlations and complex factor loadings in both INA and DPA factors. Thus, it was reported that this item may simultaneously reflect both DPA and INA as demonstrated by cross-loaded factor loadings in the exploratory analysis. Collectively, these findings suggest that a single-item measure of effort may not fully capture a gendered depression experience, particularly for Black men.

Scholars point to more descriptive measures of effort in the context of the Black experience, such as John Henryism, which is characterized as an “individual’s self-perception that he can meet the demands of his environment through hard work and determination” (James, 1994; James, Hartnett, & Kalsbeek, 1983). This high-effort and active coping style has been described as the belief that hard work and persistent effort will allow Black men to overcome the demands of their environment. These findings, coupled with extant literature, suggest that perceptions of effort may be indicative of a more complex psychosocial experience and, thus, may be more sensitive to the varying contexts in which Black men live, work, and play. This assertion warrants further investigations in future studies to determine the extent to which perceived effort is reflective of a more multifaceted and dynamic construct for this population.

EFA results revealed two emergent factors associated with depressive symptomatology. These findings diverge from previous literature using an exploratory approach among older Black men in clinical settings (Callahan & Wolinsky, 1994; Love & Love, 2006). In the current study, the hypothesized depressed affect and interpersonal factors merged into a single factor reflecting both constructs. This specified structure was confirmed to have the best fit in the CFA of the 11-item scale in comparison to the a priori three-factor model structure found in a previous study (Assari & Moazen-Zadeh, 2016). Collectively, these findings provide additional support that Black men in community-based settings experience depressive symptomatology that is distinct from that of their male counterparts in other contextual settings.

There are limitations that may influence the interpretation of results. First, the use of the 12-item CES-D scale excludes symptomatology related to broader somatic changes (e.g., weight gain, appetite change) which are reflected in the original 20-item scale. Studies report that Black Americans may report more somatic complaints related to depression in the clinical setting (Baker, Okwumabua, Philipose, & Wong, 1996). Yet, less is known regarding the role of somatization using population-based measures. Future studies are needed to extend findings to the full CES-D measure to assess whether higher endorsement of somatic symptoms is evident among Black men in community-based samples. In addition, the community-based sampling approach yielded a subset of Black men who were relatively young, had obtained a high school degree or higher, and were employed. Demographics were most likely driven by the type of settings that were frequented during the data collection period, such as barbershops and HBCUs. The demographic composition may have also contributed to the presence of high depressive symptomatology that was found in over half of the study sample as well as the high CES-D mean score. In fact, studies report that young and single Black men are at a greater risk for depression due to limited social support systems among peers or romantic relationships (Jones-Webb & Snowden, 1993; Neighbors & Howard, 1987). Future studies should explore the presence of measurement invariance of the CES-D to further disentangle differences in symptomatology shaped by demographic factors such as age, education, and employment status.

Despite these limitations, this study is an important contribution toward further understanding heterogeneity in Black men’s depression. Although there is large body of evidence that examines depression among Black Americans, this study builds on literature assessing the factor structure of a widely used scale by introducing key psychometric and dimensional differences in CES-D item functioning that are unique to community-dwelling Black men. Data collection procedures used in this study also highlight critical points of interaction for future community-based research focused on Black men. A noted strength of this approach, compared with previous factor analysis studies, is the use of data collection locations that are culturally specific to the Black men (e.g., barbershops and HBCUs) and trusted community partners (e.g., barbers). Extant literature highlights these points of interaction as important features of improving survey implementation (Cowart et al., 2004; Hart & Bowen, 2004; Luque, Ross, & Gwede, 2014; Releford et al., 2010). Compared to other studies focused in the clinical setting, this approach may yield a more relaxed atmosphere in which emotional disclosure, such as the presence of depressive symptoms, can be more easily discussed. Yet, the impact of this outreach strategy has not yet been extensively applied to community-based research in the mental health field. This study highlights an opportunity for the meaningful inclusion of trusted community liaisons as partners in cultivating mental well-being among Black men. Future studies should consider similar engagement strategies as detailed in this study as a launching point for creating effective mental health promotion efforts.

These results provide important insights as to how Black men may exhibit and endorse particular depressive symptoms within the general population. Clinicians and public health professionals building engagement strategies with Black men with depressive symptoms may benefit from a multifaceted approach, using different therapeutic or interpersonal approaches for each dimension of depression illustrated in this study. Moreover, researchers gathering evidence on etiological factors of depression should emphasize the unique social factors at play in the lives of Black men, such as discrimination, criminal justice involvement, and diminished upward mobility, and how these factors differentially contribute to depressive symptom clusters in this population. Finally, these findings present an opportunity to extend researchers’ knowledge of depression to identify drivers of known physical health disparities in this population (Hawkins, Watkins, Bonner, & Thompson, 2016; Lustman et al., 2000; Melin, Thunander, Svensson, Landin-Olsson, & Thulesius, 2013). Researchers incorporating these findings into their work should address relationships between depressive symptoms, at the factor level, and their relationship to deleterious health behaviors in order to develop varied strategies for disease prevention. To this end, increased collaborations between mental health providers and public health professionals are needed to shift the trajectory of depression research toward a prevention framework and present uncharted opportunities for reshaping mental well-being for community-dwelling Black men.
